# Boosting 2-photon vision with adaptive optics

**DOI:** 10.1167/jov.23.12.4

**Published:** 2023-10-06

**Authors:** Hannah K. Doyle, Sofie R. Herbeck, Alexandra E. Boehm, John E. Vanston, Ren Ng, William S. Tuten, Austin Roorda

**Affiliations:** 1 Department of Electrical Engineering and Computer Sciences, University of California Berkeley, Berkeley, CA, USA; 2 Herbert Wertheim School of Optometry and Vision Science, University of California Berkeley, Berkeley, CA, USA

**Keywords:** adaptive optics, scanning light ophthalmoscopy, 2-photon vision, photoreceptors, psychophysics

## Abstract

The 2-photon effect in vision occurs when two photons of the same wavelength are absorbed by cone photopigment in the retina and create a visual sensation matching the appearance of light close to half their wavelength. This effect is especially salient for infrared light, where humans are mostly insensitive to 1-photon isomerizations and thus any perception is dominated by 2-photon isomerizations. This phenomenon can be made more readily visible using short-pulsed lasers, which increase the likelihood of 2-photon excitation by making photon arrivals at the retina more concentrated in time. Adaptive optics provides another avenue for enhancing the 2-photon effect by focusing light more tightly at the retina, thereby increasing the spatial concentration of incident photons. This article makes three contributions. First, we demonstrate through color-matching experiments that an adaptive optics correction can provide a 25-fold increase in the luminance of the 2-photon effect—a boost equivalent to reducing pulse width by 96%. Second, we provide image-based evidence that the 2-photon effect occurs at the photoreceptor level. Third, we use our results to compute the specifications for a system that could utilize 2-photon vision and adaptive optics to image and stimulate the retina using a single infrared wavelength and reach luminance levels comparable to conventional displays.

## Introduction

It is widely thought that humans are only sensitive to the portion of the electromagnetic spectrum between 390 and 830-nm. In fact, sensitivity functions for the three cone types in the retina are only defined on this range (Stockman, Sharpe, & Fach, 1999; Stockman & Sharpe, 2000). However, human sensitivity to infrared light has been observed since the mid-20th century (Griffin, Hubbard, & Wald, 1947; Walraven & Leebeek, 1963; Vasilenko, Chebotaev, & Troitskii, 1965; Sliney, Wangemann, Franks, & Wolbarsht, 1976; Dmitriev et al., 1979; Theodossiou, Georgiou, Hovhannisyan, & Yova, 2001). This infrared light also did not appear *red* when observed; rather, many noted that it was similar in appearance to light at half its wavelength. This observation pointed to a 2-photon process occurring somewhere in the eye. Some hypothesized that second harmonic generation was taking place at the cornea, generating photons of visible light that could then be detected at the retina (Zaidi & Pokorny, 1988). More recently, it was shown through electrophysiological experiments that 2-photon vision is the result of direct photoisomerization in the chromophores (Palczewska et al., 2014).

The process of 2-photon absorption has been well characterized for its use in fluorescence microscopy. This phenomenon requires 2 photons to arrive at the same excitable molecule simultaneously. Thus, it depends greatly on the spatiotemporal density of incoming photons. Under diffraction-limited conditions, the number of 2-photon absorptions $n_{a}$ generated when using a pulsed laser is dictated by

(1) $$\begin{array}{c} n_{a}\propto \frac{p_{0}^{2}\;N\;A^{4}}{\tau_{p}}\; \end{array}$$

where $p_{0}$ is the average laser power, NA is the numerical aperture of the focusing element, and $\tau_{p}$ is the pulse duration (Denk, Strickler, & Webb, 1990). Thus, there are multiple avenues for boosting the number of 2-photon absorptions in order to make the 2-photon effect more visible. Because the uncorrected human eye does not typically have diffraction-limited optics, prior work has focused on enhancing 2-photon vision by decreasing $\tau_{p}$ through the use of short-pulsed lasers or by increasing the laser power $p_{0}$.

Fortunately, adaptive optics (AO) has matured to the level where we can correct the eye’s aberrations, allowing us to leverage the numerical aperture parameter NA (Liang, Williams, & Miller, 1997). AO allows us to use the largest physiological pupil sizes and achieve high spatial concentration of photons at each photoreceptor, effectively increasing NA and further enhancing the 2-photon effect, so that the same light can be made visible with AO while being almost invisible without it.

Others have probed characteristics of 2-photon vision, including acuity (Artal, Manzanera, Komar, Gambín-Regadera, & Wojtkowski, 2017) and contrast sensitivity (Kaczko et al., 2022), as well as the factors influencing it such as pulse duration (Marzejon, Kornaszewski, Wojtkowski, & Komar, 2021). However, the benefits of an AO correction on 2-photon vision have not yet been studied. In order to characterize these benefits, we carry out color- and luminance-matching experiments that help demonstrate the boost that AO provides in the salience of the 2-photon effect. We supplement these results with predictions about AO’s enhancement of 2-photon vision, using a simple model of light capture by a hexagonal mosaic both with and without aberrations present. We are also able to collect images of the retina at varying levels of defocus that we combine with our perceptual results to establish a link between the best focus for the photoreceptors and the brightest 2-photon effect, building on the work done in (Gorczynska I, et al. *IOVS* 2022;63:ARVO E-Abstract 4447—F0126) and (Doyle H, et al. *IOVS* 2022;63:ARVO E-Abstract 4551–F0465).

Since its discovery, 2-photon vision has generated interest for its use in clinical applications such as microperimetry for testing visual function (Ruminski et al., 2019; Łabuz et al., 2020; Łabuz et al., 2021; Wei et al., 2021; Mehta, Palczewska, Lin, & Browne, 2022; Marzejon, Kornaszewski, Bogusławski, et al., 2021). Because infrared light is less prone than visible light to scattering from opacities in the eye’s optics, the 2-photon effect is potentially useful for testing visual function in aging or precataractous eyes.

Our interest in 2-photon vision is for the entirely separate application of display technology, especially for displays that simultaneously image and stimulate the retina (Harmening, Tuten, Roorda, & Sincich, 2014; Sabesan, Schmidt, Tuten, & Roorda, 2016). Such displays typically image using barely visible infrared light and stimulate simultaneously using visible wavelengths. The infrared images of the retina are often used to inform the delivery of gaze-contingent stimuli at visible wavelengths. This multiwavelength operation is susceptible to errors introduced by transverse and longitudinal chromatic aberrations. Both must be corrected in order to deliver light at visible wavelengths to precise locations on the retina when using an infrared image for tracking (Harmening, Tiruveedhula, Roorda, & Sincich, 2012). However, the 2-photon vision phenomenon means that infrared wavelengths can be made visible under certain conditions and invisible otherwise. This means that a display could be conceived that uses the same infrared wavelength for both invisible imaging and visible stimulation, thereby eliminating the aforementioned chromatic aberration challenges associated with the current paradigm. We use the results from our color- and luminance-matching experiments to propose a system that uses AO combined with short pulses to achieve 2-photon stimulation with a luminance that is similar to conventional displays while satisfying ANSI standards for safe power levels at the eye.

## Methods

The human subjects protocol was reviewed and approved by the University of California, Berkeley Institutional Review Board and adhered to the tenets of the Declaration of Helsinki regarding ethical treatment of human subjects for research. Six subjects participated in total, four of whom are coauthors of this article. Each provided informed consent before participating. All subjects self-reported to have no ocular disease or condition that might affect imaging. All subjects were dilated and cyclopleged using one drop of 1% tropicamide and one drop of 2.5% phenylephrine. For all experiments reported here, light exposure levels were kept within the maximum exposure limits as specified by the Z136.1 ANSI standard for the Safe Use of Lasers (ANSI, 2014). Detailed light safety calculations are provided in Appendix A.

We carried out experiments using an adaptive optics scanning light ophthalmoscope (AOSLO). More details of the system are provided in other studies (Roorda et al., 2002; Wang et al., 2019; Mozaffari, LaRocca, Jaedicke, Tiruveedhula, & Roorda, 2020), and so only the most relevant aspects of the system will be described here. In the AOSLO, light was delivered in a raster-scanned pattern over a 0.9${}^{\circ }$ × 0.9${}^{\circ }$ square field across the retina. For the experiments done at 1,064-nm, a ∼60-Hz frame rate with 256 lines per frame was achieved with a combination of a slow galvanometer scanner at 60 Hz and a fast resonant scanner at ∼16 kHz. For experiments done at 940-nm, the slow galvanometer scanner was operating at 30 Hz for a ∼30-Hz overall frame rate. The fast resonant scanner operates in a sinusoidal fashion, which causes it to move more slowly at the left and right edges of the raster, resulting in brighter bands at those edges. In order to achieve a more uniformly bright appearance for ease of color matching, we centered a beam block at the retinal plane in the optical path, which eliminated these bright edges. The focused spot was corrected to near diffraction-limited using AO, which is a set of optical methods to measure and compensate ocular aberrations (Liang et al., 1997). In this system, the wave aberrations were measured with a custom-built Shack–Hartmann wavefront sensor, and the aberrations were corrected with a 97-actuator deformable mirror (DM97-08; ALPAO, Montbonnot-Saint-Martin, France). The AO ran continuously in closed loop during all AO-corrected conditions. For AO-off conditions, the deformable mirror was set to a precalibrated “flat-state.” Defocus offsets were added using the same deformable mirror from the forementioned AO-corrected or flat-states.

The current AOSLO can operate with up to four wavelengths scanning independently or simultaneously. All light sources were drawn from a supercontinuum laser (SuperK Extreme; NKT Photonics, Birkerod, Denmark) and coupled into four single-mode fibers. Specific wavelengths for each channel were selected by placing spectral filters into the optical path prior to fiber coupling. For the experiments reported here, narrowband filters centered at either 1,055-nm [full width at half maximum band width (FWHM BW) = 85.5-nm] or 940-nm [FWHM BW = 21.9-nm] were used in the first channel for 2-photon stimulation (reasons for using these wavelengths are given in later sections). Although we used a filter that is centered at 1,055-nm, the spectrum of our supercontinuum light source contains a peak at 1,064-nm, so the transmitted light was dominated by 1,064-nm light. Wavefront sensing was also done with the same 940- or 1,064-nm wavelengths. A second near-infrared channel with light centered at 840-nm [FWHM BW = 21.1-nm] was used to facilitate tracking and imaging (see below). The final two channels had wavelengths centered at 532 [FWHM BW = 23.9-nm] and 680-nm [FWHM BW = 29.8-nm] and were used for color matching. The power of each visible channel could be adjusted using computer-controlled acousto-optic modulators. The light in all four channels could be shut off completely using electronic shutters in their respective optical paths.

The light from the supercontinuum laser comprises short “chirped” pulses at 100 MHz. The pulse width depends on the wavelength and the bandwidth and was measured experimentally to be 14.4 ps for the 1,064-nm waveband and 8.0 ps for the 940-nm waveband. These pulses, although quite long relative to the femtosecond-scale pulse widths used for 2-photon fluorescence imaging, were short enough to elicit 2-photon vision, especially with the addition of AO.

### Experiments showing defocus dependence, power dependence, and AO boost (1,064-nm)

**Figure 1.**
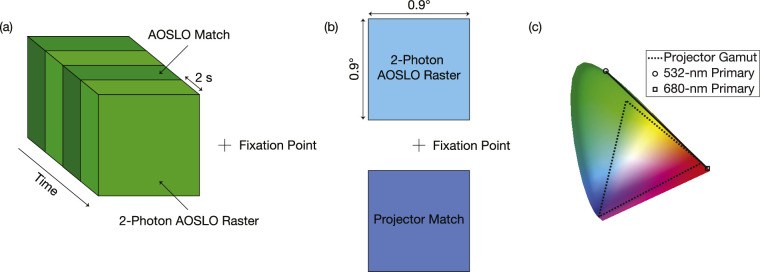
Color-matching experiment details. (a) The 1,064-nm 2-photon AOSLO raster alternates in time with an AOSLO-generated matching raster. The matching raster is a mixture of 532- and 680-nm light, which subjects tune until it is indistinguishable from the 2-photon raster. (b) The 940-nm 2-photon AOSLO raster is a 0.9${}^{\circ }$ × 0.9${}^{\circ }$ field positioned 0.675${}^{\circ }$ above fixation. Subjects tune the color of an equally sized projector-generated square that is positioned opposite their fixation in order to achieve a match to the appearance of the 2-photon raster. (c) The 532-nm and 680-nm primaries used in 1,064-nm experiments are shown on the chromaticity diagram. The line connecting them represents all possible mixtures of the two. The gamut of the projector used for matching in 940-nm experiments is also shown, where the three vertices of the triangle correspond to its red, green, and blue primaries.

The first set of experiments was done using light centered at 1,064-nm. This wavelength was chosen for two reasons. First, our supercontinuum light source had a lot of available power at this band, which enabled a measurement of the power-squared dependency of the 2-photon effect. Second, the peak spectral sensitivity of the eye lies close to half of this wavelength, making the 2-photon effect especially visible. To match color and luminance, subjects used a combination of 532-nm and 680-nm light, which was also delivered using the AOSLO (see above). Thus, the 2-photon field and matching fields were positioned at the same point in space and were alternated in time as depicted in Figure 1a. Electronically controlled mechanical shutters were used to switch between the two fields every 2 seconds while the subject adjusted the mixture of 532- and 680-nm light in order to achieve a match to the 1,064-nm raster. Subjects adjusted the relative proportions of 532- and 680-nm light using the motorized faders on a MIDI controller (Behringer X-Touch Compact, Willich, Germany). To obtain quantitative measures of the luminance for the matches, we multiplied the measured total power of the 532- and 680-nm sources by the fractional intensity levels chosen in a given match and computed equivalent luminance considering the field size on the retina using methods described in the appendix of Domdei et al. (2018).

Through-focus profiles of the 2-photon effect were collected by instructing subjects to make color and luminance matches to the IR raster pattern for a range of defocus settings, which were set by adding defocus offsets to the deformable mirror. The defocus settings were not uniform but were more closely spaced around best focus to capture the maximum response in greater detail. This “best focus” was estimated prior to the beginning of the experiment, using a procedure where subjects adjusted defocus using a dial on the MIDI controller to maximize the luminance of the 2-photon effect. Each subject made three color matches at 11 defocus levels, which were presented in a pseudorandom order. The power at 1,064-nm for these experiments ranged from 921 to 927 µW for four of five subjects. For the fifth subject, 20230R, the power was 837 µW due to technical limitations.

The power dependence of the 2-photon effect and the boost due to AO were measured by instructing subjects to make color and luminance matches for a range of five uniformly distributed power settings ranging from 81 µW to 795 µW at the eye. These power levels were achieved using neutral density filters and presented in a pseudorandom order. For these experiments, subjects first adjusted the defocus offset added to the deformable mirror to optimize for the luminance of the 2-photon effect both with and without AO. These two defocus settings were then maintained during the subsequent matching.

### Experiments confirming that 2-photon effect occurs at the photoreceptor layer (940-nm)

The second set of experiments was done with light centered at 940-nm, chosen because there were detectors commercially available to perform imaging along with the color and luminance matching. Subjects matched to the 0.9${}^{\circ }$ AOSLO raster using a similar-sized square generated by a DLP LightCrafter projector (Wintech, Carlsbad, CA, USA) that was coaligned with the AOSLO. The projector display had a total field of view of ∼18 degrees and was viewed through a Badal optometer configuration, which enabled the defocus setting for each individual to be adjusted and for which all the light from the projector was delivered into the eye via a ∼7-mm exit pupil that was coaligned with the exit pupil of the AOSLO (this is often referred to as a Maxwellian view). The projector display was programmed using MATLAB (MathWorks, Natick, MA, USA) with the Psychophysics Toolbox (Brainard, 1997; Pelli, 1997; Kleiner et al., 2007) to present calibrated RGB images to the subject. In this experiment, subjects saw two squares positioned on opposite sides of a fixation cross, as shown in Figure 1b. The fixation cross and matching square were generated by the projector, and the 2-photon square was generated by the AOSLO’s raster scan. Subjects adjusted the RGB values of their projector-generated matching square using the motorized faders on the MIDI Controller.

The projector display was configured in Maxwellian view, making direct measurements of its radiometric properties challenging. To obtain quantitative measures of the luminance for the projector matches, we first used a spectroradiometer (PR650, PhotoResearch [now Jadak], North Syracuse, NY, USA) to measure the shape of each primary’s spectral power distribution. We then empirically measured the spectral responsivity of our optical power meter (Newport, Irvine, CA, USA) and used that power meter to measure the power of each primary at its maximum intensity setting. Using these measurements, we could infer the scaling factor, which converted the spectra measured by the spectroradiometer into absolute units of power. Taking into account the field size on the retina, we could then compute the luminance of any linear combination of the projector’s primaries.

At 940-nm, we also wished to collect images of the subjects’ retina. To do this, we placed a 90:10 beamsplitter in the path of the wavefront sensor and collected the diverted light using an avalanche photodiode (APD410A/M; Thorlabs, Newton, NJ, USA) in order to generate an image. Because the power was low and the sensitivity of the detector was not ideal, we needed to average several 940-nm images together in order to achieve a high enough signal-to-noise ratio for the image. To facilitate frame averaging, we simultaneously tracked the eye motion by imaging at 840-nm and used this to stabilize the incoming 940-nm images for averaging. As a result, we were limited in the defocus range that we could image over, because we lost signal in 840-nm as we went more out-of-focus and could no longer track. As the 840-nm light used for imaging was readily visible, the imaging was done alternately but not simultaneously with the color and luminance matching. During the color and luminance matching, an electronic shutter was used to block the 840-nm channel entirely.

All 940-nm experiments were done with AO correction. As in the 1,064-nm experiments, varying levels of defocus were added to the deformable mirror in order to collect a through-focus profile of the 2-photon effect where the spatial confinement of the raster-scanned spot was varied. Each subject made three color matches at 11 defocus levels, which were presented in a pseudorandom order. The power at 940-nm ranged from 206 to 209 µW across five sessions.

### Modeling

To confirm that the results were consistent with expectations, we performed simple modeling to predict the increases in 2-photon luminance that we would expect with defocus and aberration correction via AO. We modeled this using custom code written in MATLAB.

In this model, we iterated through defocus levels ranging from −1 D to 1 D in increments of 0.01 D. At each defocus, we computed the point spread function (PSF) of 1,064-nm light at the retina resulting from the added defocus combined with the aberrations of the eye. After the PSF was computed, we applied a binary mask intended to mimic light collection by a cone mosaic. This mask consisted of cones that were modeled as circles with a radius of 0.5 arcmin in a close-packed hexagonal arrangement that extended over the entire field size of the simulation, which was 66.9 arcmin. Because the number of 2-photon absorptions increases with the square of light intensity as dictated by Equation 1, we took the squares of the integrated PSF intensity for all cone apertures in the mask and summed them to produce a proxy for 2-photon luminance.

In order to evaluate the boost in 2-photon luminance provided by an AO correction, we ran this model for an ideal, aberration-free eye and compared it to results using real aberration data. We used Zernike coefficients from 10 deidentified adult human subjects (mean age: 26.3 ± 4.3 years; range: 22–34 years) with a 7-mm or greater pupil size. These subjects were taken from a previously published dataset that included 74 human eyes (Cheng et al., 2004). For the purposes of our modeling, the 10 subjects were uniformly drawn from the larger dataset ordered by high-order root-mean-square (RMS) aberration, avoiding the extremes.

## Results

### Human experiments

Color- and luminance-matching results for the 1,064-nm and 940-nm experiments described above are presented in order below.

#### At 1,064-nm

**Figure 2.**
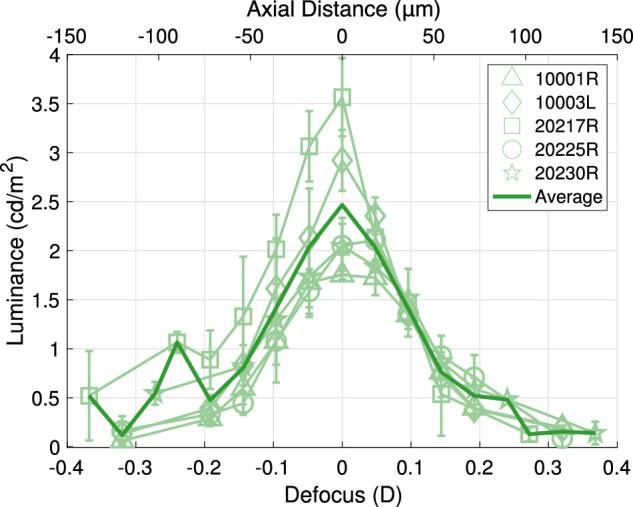
Through-focus 2-photon luminance profile at 1,064-nm. Subjects made three matches using a mixture of 532- and 680-nm light at each of the 11 defocus levels. The total luminances of their matches are plotted against defocus and axial distance in microns. We fit a Gaussian to each subject's data and center the closest sample to the Gaussian's peak at 0 D for ease of comparison between subjects. The matched luminance of the 2-photon effect peaks around an optimal focus for each subject.

The through-focus luminance profiles of the 2-photon effect at 1,064-nm for each subject are shown in Figure 2. For these experiments, the amount of 1-photon isomerization of 1,064-nm light is negligible—the 680-nm luminance decreases to 0.018 cd/m${}^{2}$ on either defocus extreme, on average. The 680-nm component also rises with the 532-luminance component as the focus changes, suggesting that both are necessary in order to match the color appearance of the defocus-dependent 2-photon effect. Thus, we plot the combined luminance of both the 532-nm and 680-nm components in Figure 2 to represent the 2-photon effect’s total luminance.

We can calculate the equivalent wavelength that is metameric to each match combination, as we know the proportions of 532- and 680-nm light along with their absolute power levels. In doing so, we find that the average through-focus equivalent monochromatic wavelengths are 542-nm, 544-nm, 553-nm, 554-nm, and 558-nm across our five subjects. Thus, the color appearance of the 2-photon effect at 1,064-nm tends to skew toward wavelengths that are longer than the expected half-wavelength of 532-nm. In these results, we also see an increase in the luminance of the 2-photon effect around an optimal focus for each subject. We will later show through imaging results at 940-nm that this optimal focus corresponds to the defocus setting where light is focused most tightly at the layer of the photoreceptors.

**Figure 3.**
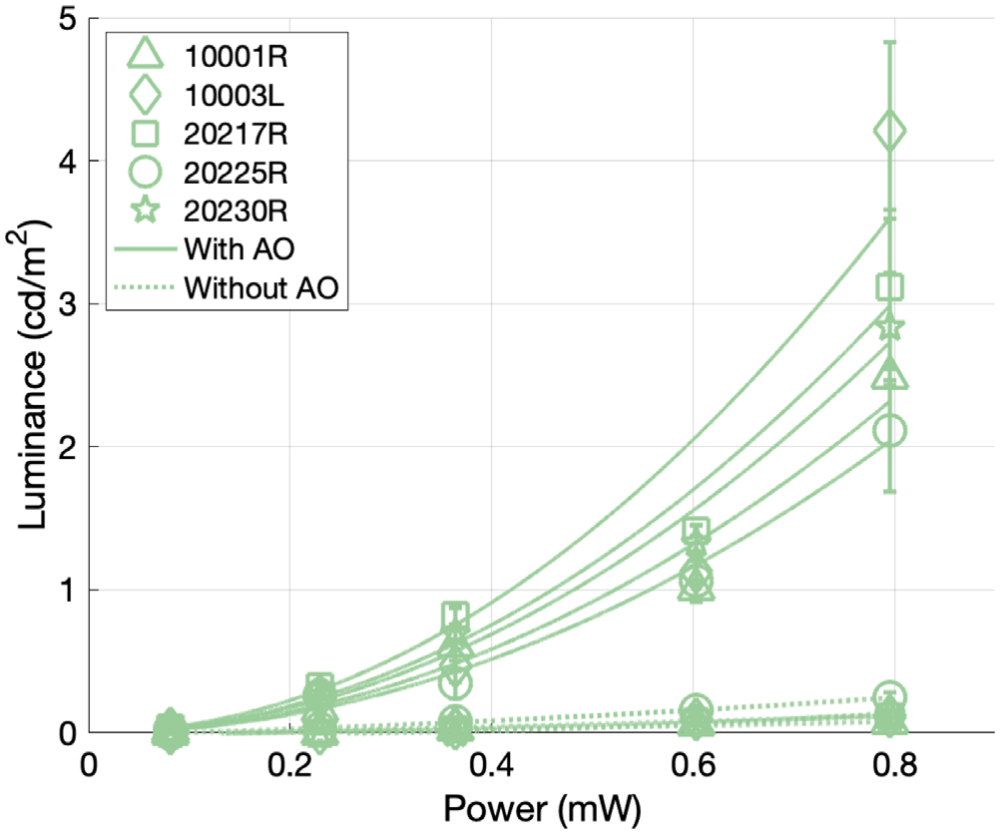
Power dependence of 2-photon luminance with and without an AO correction. Subjects made three matches with AO and three matches without AO to the 1,064-nm 2-photon raster at five laser power settings. The total luminances of their matches are plotted against laser power. We fit a quadratic model to the luminance results with and without an AO correction. The matching results show a significant boost in 2-photon luminance as a result of AO, with the boost for each subject ranging from 8.5× to 36.8× at the highest power setting.

We also investigated the power dependence of the 2-photon perceived luminance with and without AO at 1,064-nm. Subjects again matched by tuning a mixture of 532- and 680-nm light, and the total luminance of their matches at five power levels is plotted in Figure 3. We see that the luminance of the 2-photon effect varies with the square of the laser power, as expected. For all subjects, there is a significant boost in luminance when AO is used. This boost ranges from 8.5× to 36.8× at the highest power level, with an average increase of 25.5× when using AO. These results establish conditions under which nearly invisible infrared light can be made visible through the use of AO alone.

#### At 940-nm

**Figure 4.**
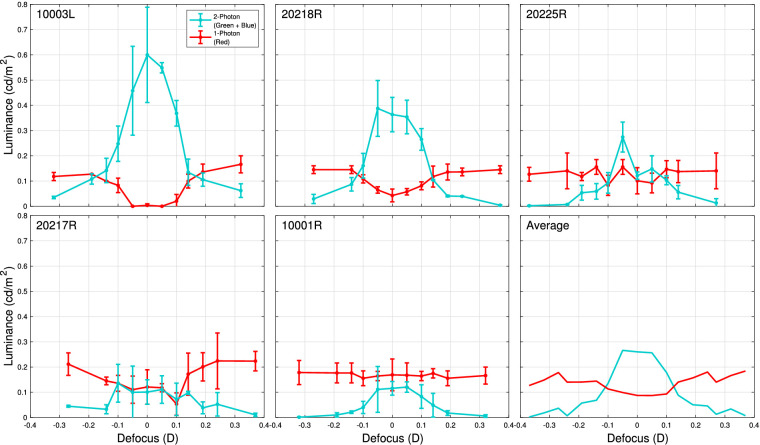
Through-focus 2-photon luminance profile at 940-nm. Subjects made three matches using an RGB projector to the 940-nm 2-photon raster at 11 defocus levels. The red component and the combination of the green and blue components of their matches are plotted as luminance for each defocus. The green and blue mixture is taken to represent the luminance of the 2-photon effect at 940-nm, which increases around an optimal focus for each subject. Each subject’s data are aligned for comparison using the same procedure as in Figure 2.

For experiments at 940-nm, there is a nonnegligible visible red component due to 1-photon isomerizations of 940-nm light in addition to the 2-photon effect. Thus, in Figure 4, we separate this 1-photon component from the 2-photon component of the match made using an RGB projector. The 1-photon isomerizations should appear to be a pure red as they would only stimulate long-wavelength sensitive cones, so we take the red value of the RGB match to represent the 1-photon component entirely. The fact that the red luminance persists and remains relatively flat and consistent between subjects at the limits of the defocus range is evidence that the red primary encodes 1-photon isomerizations. The 2-photon component can then be explained by the remaining combination of the green and blue projector primaries. These RGB values are converted to luminance and plotted in Figure 4 at each of the 11 defocus levels. We again see that there is an optimal defocus level around which the luminance of the 2-photon effect peaks for each subject, as it did in our experiments at 1,064-nm.

It is also apparent that the red component appears to be anticorrelated with the green and blue components, especially for subjects 10003L and 20218R. That is, there is a decrease in the red component around defocus values where the 2-photon component is dominant. This cannot be due to a reduction in 1-photon isomerizations. Rather, we surmise that it is because matching was done using a projector, and subjects were limited in the gamut of colors that could be achieved with the matching stimulus, as shown in Figure 1c. As a result, some subjects could not achieve the same saturation on the matching stimulus as they saw in the 2-photon stimulus and instead opted to decrease the red component of their RGB match in an attempt to compensate, at some cost to the fidelity of the overall match.

**Figure 5.**
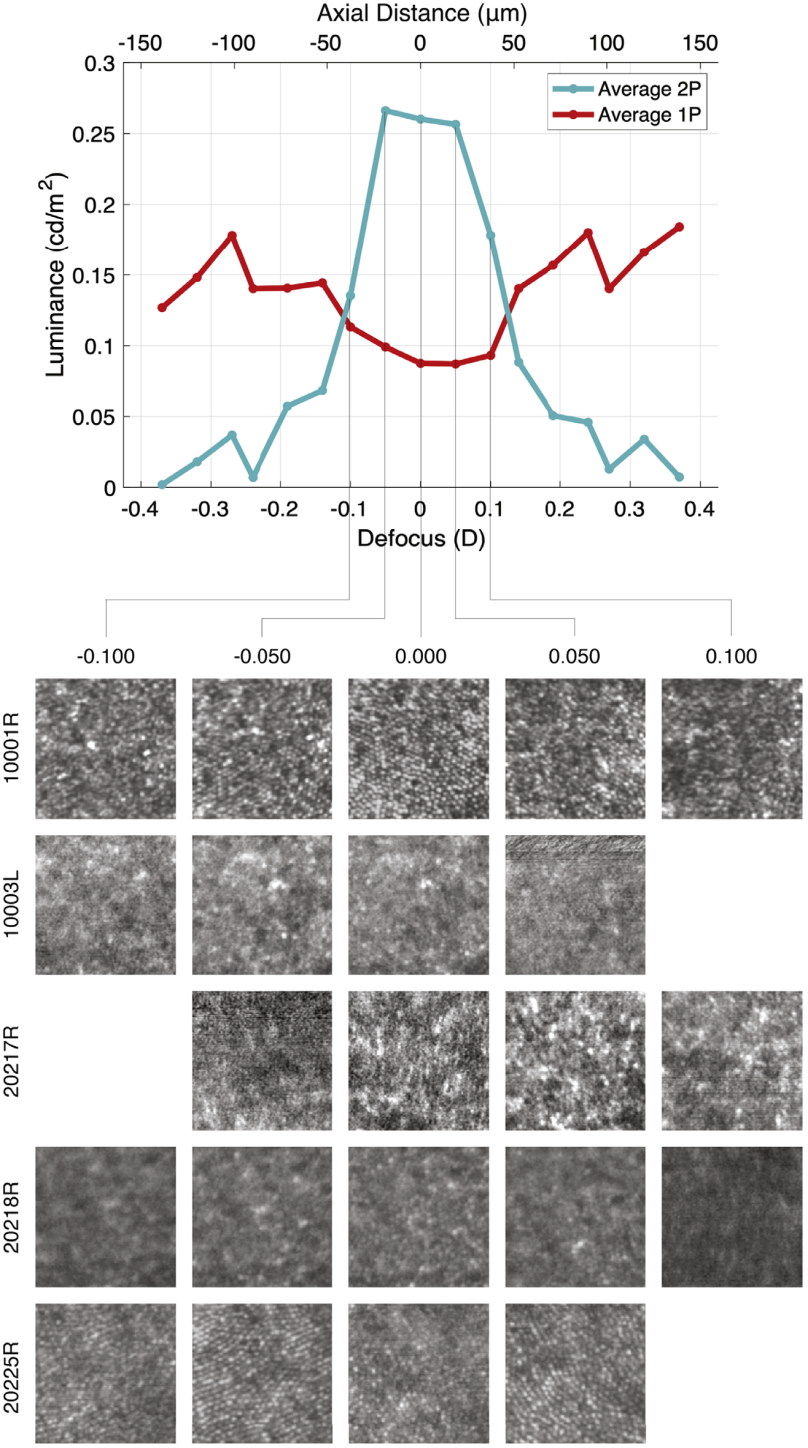
Imaging results and corresponding 2-photon luminance data at 940-nm. Images were collected at the defocus levels where subjects made matches. The central thirds of each subject’s images are shown in separate rows, with each column corresponding to a given defocus level. Lines connect each of the defocus levels to the corresponding point in the plot of average 2-photon luminance across all subjects. The sharpest images of the photoreceptors tend to coincide with the brightest matches.

The use of AO also made it possible to collect images at 940-nm with the same channel that was used for 2-photon stimulation. For each subject, we collected images for at least four defocus settings around the best focus. In Figure 5, we compare the images collected at each defocus level to the corresponding average match luminances across all subjects. We see that the optimal focus for maximizing 2-photon luminance is at or near the optimal focus for sharp images of the photoreceptors. This correlation is more apparent for some subjects than others; as noted before, we relied on simultaneous tracking using 840-nm imaging in order to frame-average images in 940-nm due to low signal and low photodetector sensitivity to 940-nm. Unstable tracking could cause the 940-nm image signal to noise ratio (SNR) to suffer, or it may have prevented us from collecting an image entirely, as is the case for subjects 10003L, 20217R, and 20225R at certain defocus levels. Nevertheless, we do tend to see sharper photoreceptor structure in images near the defocus level where 2-photon luminance is highest. Indeed, the imaging results suggest that the optimal focus for imaging the cones is at least within ∼25 microns axially from the optimal focus for 2-photon luminance. That is, the 2-photon luminance is increased when light is focused near the photoreceptors. This provides imaging-based support for the finding that the 2-photon absorptions are occurring at the photoreceptors and that 2-photon vision is the result of direct 2-photon isomerization of cone photopigments.

### Modeling

**Figure 6.**
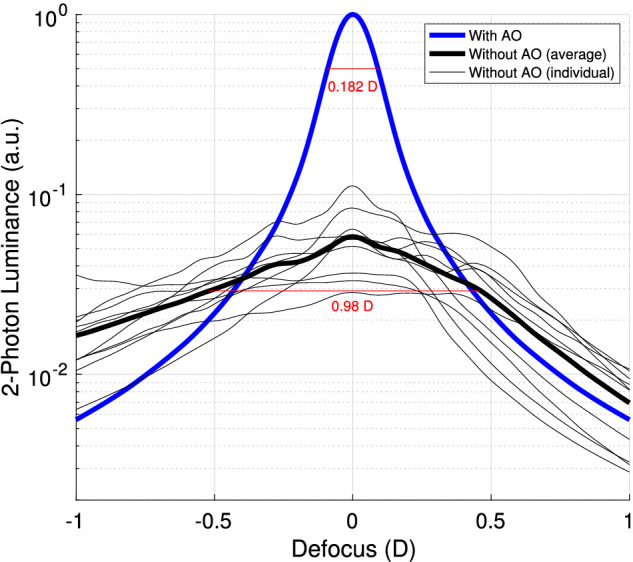
Modeling of through-focus 2-photon luminance profile and boost from AO. The blue line is the simulated 2-photon luminance on a log scale (arbitrary units) versus defocus for a perfect AO correction. The thin black lines are the simulated 2-photon luminances for 10 eyes with varying types and degrees of aberration. The thick black line is the average of these uncorrected eyes. All modeling results are normalized so that the ideal eye peaks at 1 a.u. and aligned so that all peaks are at 0 D. The FWHMs of the with- and without-AO plots are labeled in red.

The results of our modeling for the luminance of the 1,064-nm 2-photon luminance are shown in Figure 6. There is a significant boost in the modeled 2-photon luminance of the AO-corrected ideal eye over the aberrated eyes. This boost ranges from 9.0× to 35.1× between the peaks of the aberrated eyes and the ideal eye, with an average of 20.1×. For comparison, the average boost from AO found in our experiments was 25.5×, ranging from 8.5× to 36.8× among the five subjects in experiments at 1,064-nm. That the boost is slightly smaller in modeling on average may be due to our method for finding optimal focus prior to carrying out the color and luminance matching. Subjects used a dial on the MIDI controller to change the defocus setting until they found the brightest 2-photon effect both with and without AO. Because the luminance without AO was so low, subjects may not have been sensitive enough to tune the defocus to its truly optimal setting. This would have resulted in an inflated boost between the AO on/off cases relative to the modeling, where boost factors are calculated using the true optimal defocus setting for each real eye.

The full width at half maximum (FWHM) of the luminance versus defocus plot for the eye with an ideal AO correction is 0.182 D (68.3 µm). We can compare this to our own results by fitting a Gaussian to all subjects’ luminance versus defocus data from Figures 2 and 4. In doing this, we find that the FWHM of our subjects’ data is 0.243 D (91.1 µm) and 0.221 D (83.0 µm) for 1,064-nm and 940-nm, respectively. For comparison, the FWHM of the average non-AO-corrected eye’s luminance versus defocus in our modeling was found to be 0.980 D (367.5 µm). Thus, the FWHM values found in our data agree more closely with the modeled luminance under an AO correction, as expected.

## Discussion

### Future system specifications

We have shown that AO can be used to create a strong 2-photon effect without the need for ultrashort, femtosecond-scale pulses. A system that combines short pulses with AO would then reach even higher luminance levels that are comparable to conventional displays. Our results establish a benchmark and can be used to inform the specifications for the development of displays based on 2-photon vision, which would eliminate the chromatic aberration challenges associated with gaze-contingent displays that image with infrared (IR) and stimulate with visible light. If we take the average luminance at the highest laser power from Figure 3, we can extrapolate using the quadratic power dependence to determine the power required to achieve a desired display luminance. Combining this with a reduction in pulse duration using Equation 1, we can reach even higher luminances with reduced power. In doing so, our data suggest that a 500-cd/m${}^{2}$ display could be achieved with an average power of 0.86 mW and a pulse duration of 100 fs with the addition of an AO correction. Several candidate lasers for such a system already exist, with wavelengths close to 1,064-nm and pulse widths close to 100 fs (NKT Photonics, 2022; Menlo Systems, 2023; ThorLabs, Inc., 2023). In such a system, the femtosecond source would be used for stimulation, appearing like a monochromatic source around half of its IR wavelength. A continuous-wave source at the same IR wavelength would then serve as the imaging light while remaining nearly invisible due to its low temporal concentration. This imaging and stimulating system would enable precise, single-cone level retinal stimulation without the careful correction of chromatic aberration that would typically be required. In implementing such a system, we must also ensure that exposures are kept at safe levels. Relevant light safety calculations are outlined in Appendix A.

Performing experiments in the AOSLO system also allowed us to image using the same wavelength channel that was creating the 2-photon effect, so that retinal imaging could be done along with our psychophysical experiments. We were thus able to show that the 2-photon effect’s luminance increased when light was focused at the photoreceptors, providing further support for the finding that 2-photon vision is a result of direct excitation of cone photopigments. This result is an added benefit for any future system that combines IR imaging with 2-photon stimulation, as the optimal focus for imaging the cones will coincide with the optimal focus for precise stimulation of the cones. This is often not the case in systems that use separate wavelength channels for imaging and stimulation due to the presence of longitudinal chromatic aberrations (LCAs) in the eye’s optics, which varies across subjects. In a multiwavelength system, this intersubject variability in LCAs requires custom compensatory solutions that add complexity to system design (Jiang, Kuchenbecker, Touch, & Sabesan, 2019).

### Color appearance

In doing color-matching experiments, we found that the color appearance of the 2-photon effect at 1,064-nm was not exactly matched by 532-nm light alone, which would correspond to half of the IR wavelength. Rather, subjects found it necessary to add some amount of 680-nm light in order to achieve a match to the 2-photon effect. As discussed previously, this is unlikely to be due simply to 1-photon isomerizations of 1,064-nm light, as we found a focus dependence in the red component. That is, the added 680-nm component rose and fell in a way that was correlated with the green 532-nm component. This suggested instead that there was a redshift in the 2-photon effect’s color itself relative to the expected half-wavelength appearance.

In calculating the average through-focus equivalent wavelength, we found a mean of 550-nm and standard deviation of 6.8-nm across our five subjects. The origin of this redshift phenomenon in the 2-photon effect is unclear, but it has been observed before in color-matching experiments (Palczewska et al., 2014). One possible explanation is that the 2-photon cross section may differ between cone types, resulting in a change in the realized color appearance relative to what would be predicted under 1-photon isomerizations by light at half of the IR wavelength. If this were the case, this shift in color appearance toward redder wavelengths would suggest that L cone opsins may have a larger 2-photon cross section than M cones, thereby amplifying the L-cone activation level. Another possibility that has been suggested is that secondary absorption of slightly longer-wavelength photons emitted by a 2-photon fluorescence process in rhodopsin could lead to this redshifted color appearance (Palczewska et al., 2014).

### Individual differences

There is a notable difference between the color-matching data at 940 and 1,064-nm in the degree of intersubject variability. At 1,064-nm, there is a two fold difference in peak 2-photon luminance. This is not too surprising, as there can be intersubject variability in the quality of AO correction, scatter, or even photoreceptor coupling efficiency.

For 940-nm, however, we see a six fold difference in the highest and lowest reported 2-photon luminances. We hypothesize that this disparity could be explained by variation in macular pigment concentrations between subjects. Although macular pigment concentrations can vary by a factor of >10 across the population (Celentano, Burke, & Hammond, 2002), people generally agree on the blueness of objects in the world under normal viewing conditions. As such, they must have developed a postreceptoral amplification factor that compensates for the attenuation of blue signals by their own macular pigment density, as suggested by Webster, Halen, Meyers, Winkler, and Werner (2010). However, 2-photon stimulation by 940-nm light presents a unique scenario where the incoming light is not actually attenuated by macular pigment, yet it stimulates as if it were light near 470-nm. The resulting blue signal should therefore be subject to the same adaptive factors that would affect blue light under normal viewing conditions. Since those adaptive factors vary between people, the blueness and brightness of the 2-photon effect could be amplified to different degrees perceptually. These different amplification factors could explain the wide variation across subjects who are matching to the same 2-photon stimulus. It is also consistent with the reduced intersubject variation in the experiments at 1,064-nm, which would not be affected by the same postreceptoral amplification, as 532-nm light is virtually unabsorbed by macular pigment (Bone, Landrum, & Cains, 1992). Thus, we would expect more variation between subjects in the data collected at 940-nm due to differences in their macular pigment concentrations and resulting compensatory factors. Future work will seek to validate this hypothesis by supplementing the color-matching results with objective measurements of macular pigment optical density in each subject.

## Conclusions

In prior work, the 2-photon effect in vision has been made more salient using lasers with pulses on the scale of femtoseconds. With this work, we have shown that a boost in 2-photon luminance can instead be provided by an AO correction, through its ability to focus light tightly at the level of the photoreceptors. We used color- and luminance-matching experiments at 1,064-nm and 940-nm to characterize the through-focus luminance profile and laser-power dependence of the 2-photon effect. The use of an AO correction yielded a 25-fold boost in 2-photon luminance, on average. We supplemented these results with a model of the through-focus 2-photon luminance profile with and without AO and found a predicted boost factor and FWHM that resembled our experimental results. We used the same infrared channel that generated the 2-photon visual sensations to collect images at several defocus settings, which revealed that the 2-photon effect appeared brighter as the image of the cones became sharper. This provided image-based evidence that the 2-photon effect indeed occurs in the cones.

The phenomenon of 2-photon vision has previously been explored primarily due to its potential for testing visual function in clinical settings. With this work, we have proposed a new application for 2-photon vision: a display system that can image and stimulate the retina using one IR wavelength. Such a display would have the following benefits: First, it would eliminate the need to account for the effects of chromatic aberration between channels in a multiwavelength system. Second, the power-squared dependence of the 2-photon effect means that any 2-photon stimulation would have increased contrast as compared to the background 1-photon imaging light. Third, the defocus dependence of the 2-photon effect means the optimal focus for sharp imaging would coincide with the optimal focus for stimulation luminance. We have extrapolated from our 2-photon luminance measurements in order to specify the pulse duration and power required to achieve a 500-cd/m${}^{2}$ display using 2-photon stimulation with AO and verified that such a system is achievable with current technology. Future work may involve the development and testing of this kind of display system.

## Figures and Tables

**Figure A1.**
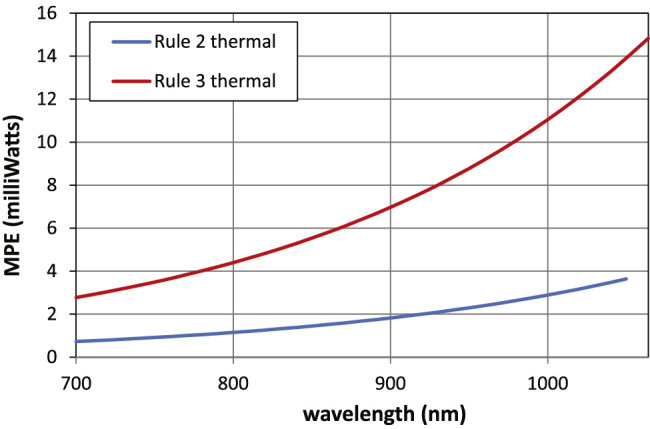
MPE as function of wavelength for Rule 2 and Rule 3 with a 0.9 × 0.9 deg field and under other specific conditions described in this article. These are for long-term, indefinite exposure times. The lower of the two is the MPE that is imposed, which, in this case, is Rule 2.

## Appendix A: Light safety considerations and calculations

### General ocular safety considerations

#### Short pulse lasers

The 2014 ANSI standard for the Safe Use of Lasers does not provide eye safety guidance for laser pulses shorter than 100 fs because of a “lack of biological data” (ANSI, 2014) (Section 8.2.2, p. 65). For pulses longer than 100 fs, the standards suggest that accumulated thermal effects are the primary hazard for long-term repeated exposure to short-pulse sources like those used for imaging. Indeed, one paper showed in a monkey retina that the light damage mechanisms for exposures to either continuous-wave or repeated 150-fs pulsed light are indistinguishable (Cain et al., 2001). Damage to S cones in a macaque retina caused by 2-photon excitation with AO has been observed (Schwarz et al., 2018). However, their exposures exceeded the ANSI maximum permissible exposure (MPE) for thermal damage, and the pulses were shorter (∼55 fs), the laser power was much higher (7 mW), and the wavelength was much shorter (730-nm) than would ever be needed for a future 2-photon display. Nevertheless, such cases need be considered before deploying a future system that employs 2-photon stimulation.

#### Infrared autofluorescence dimming

It has been previously found that there are dose-dependent reductions in the infrared autofluorescence of the retina following exposures to infrared excitation wavelengths (Masella, Williams, Fischer, Rossi, & Hunter, 2014). This “dimming” resulted from exposure levels that were below the ANSI-determined maximum permissible exposures for thermal damage and was unanticipated by the ANSI standard. Importantly, it was shown that the dose-dependent changes were the same whether the exposure was delivered with a continuous extended source, an AO-corrected raster scan, or a non-AO-corrected raster scan. In other words, they found that the changes in autofluorescence were governed by average energy and had nothing to do with the use of AO or with scanning (Masella et al., 2014). It is also important to note here that autofluorescence dimming arose from wavelengths that excited the autofluorescence, suggesting a wavelength-specific bleaching of the fluorophores. For example, Morgan et al. showed that retinal exposures to 830-nm light had no effect on 568-nm autofluorescence (Morgan et al., 2008). Given that no one has ever measured, or even suggested, that 940- or 1,064-nm light can excite autofluorescence, it is unlikely that any measurable autofluorescence and consequent autofluorescence dimming would even occur. We do not have autofluorescence capabilities in our AOSLO and so we are unable to test this. Additionally, there is no ANSI guidance for computing safety levels for any potential photochemical changes to the retina for wavelengths greater than 700-nm.

### Calculation of maximum permissible exposures for thermal damage

For this study, we considered the laser safety hazard as per the ANSI guidelines. That is, we considered the thermal damage hazard. In a raster scanning system, there are two sources of repetitive pulses that warrant consideration. First is the pulsing of the light source itself. In our case, the supercontinuum laser pulses at 100 MHz with pulse lengths for any given waveband on the order of picoseconds. This is the pulse that gives rise to the 2-photon effect. Second is the fact that at any given location in the raster, the retina is exposed to a brief pulse from the scanning beam only once per frame. According to the ANSI 2014 standard (the version of the ANSI standard that was most up-to-date during the time of this study), there are three rules to consider when using repetitive-pulse lasers. Here, we calculate the safety limits for repetitive-pulse lasers according to those rules (ANSI, 2014) (Section 8.2.3, p. 65), which are summarized below.

• Rule 1: “*The exposure from any single pulse in a train of pulses shall not exceed the MPE for a single pulse of that pulse duration.*”
  • Considering that all pulses are the same, Rule 1 is superseded by Rule 3.
• Rule 2: “*Average power MPE for thermal and photochemical hazards. The exposure from any group of pulses (or sub-group of pulses in a train) delivered in time T shall not exceed the MPE for time T.*”
  • To address Rule 2, we simply calculate the safety levels for an extended source where the light is delivered over the extent of the raster-scanned area continuously for an extended length of time.
• Rule 3: “*Multiple pulse MPE for thermal hazards. The exposure for any single pulse (t<0.25 sec) or group of pulses (T<0.25 sec), each separated by at least $t_{min}$, shall not exceed the MPE based on the width of a single pulse, or the width of a pulse group, multiplied by a multiple-pulse correction factor, $C_{P}$.*”
  • Rule 3 will necessarily be more conservative than Rule 1 since the pulse energy is constant and at a fixed frequency.

ANSI 2014 specifies the following additional factors to consider in the safety calculations:

• Intrabeam viewing, wherein all the delivered power enters the subjects' eye
• Dilation (considering that mydriatic drops are used and therefore there is no natural constriction of the pupil in response to visible light)
• The use of head and eye restraints
• Intentional viewing of the laser

ANSI 2014 generally imposes more conservative corrections to the MPEs considering all these factors.

The equations below are written so that the final MPE is in units of total average power (Watts) measurable at the surface of the cornea.

#### Application of Rule 1

*See Rule 3.*

#### Application of Rule 2

Since only near-infrared wavelengths are being used, no photochemical damage thresholds apply (ANSI, 2014).

MPE for thermal damage:

(A1) $$\begin{array}{ccc} & & \;\;\;\;\;\;\;\;\;\;\;\;\;MPE[W]\\ & & \;\;\;\;\;\;\;\;\;\;\;\;\;=\frac{1.8\cdot C_{A}\cdot C_{E}\cdot T_{2}^{-0.25}\cdot C_{pupil\_area}\times 10^{-3}}{C_{dilation\_factor}}\;\; \end{array}$$

(A2) $$C_{A}=10^{0.002(\lambda -700)},\lambda =700-1,064\;\mathrm{nm}$$

(A3) $$C_{E}=\frac{\alpha_{x}+\alpha_{y}}{2\cdot \alpha_{min}}$$

where α are the x and y dimensions of the field size in mrad and $\alpha_{min}$ = 1.5 mrad.

(A4) $$\begin{array}{c} \;\;\;\;\;\;\;\;\;\;\;\;\;C_{dilation\_factor}=\left\{\begin{array}{cc} 5 & \lambda =400-600\;\mathrm{nm}\\ 10^{0.0074(700-\lambda )} & \lambda =600-700\;\mathrm{nm}\\ 1 & \lambda >700\;\mathrm{nm} \end{array}\right. \end{array}$$

The conventional computation for $T_{2}$, which is $T_{2}=10\times 10^{\frac{\alpha -\alpha_{min}}{98.5}}$ (ANSI, 2014) (Table 6b, p. 84) where α is the angular extent of the field in radians and $\alpha_{min}$ = 1.5 mrad, does not apply for situations involving intentional viewing of the laser, such as in ophthalmoscopy applications. According to ANSI 2014 (Section 8.3.2, p. 67), “*To protect against thermally induced retinal injury, the MPE (for extended sources) shall be calculated with a thermal limiting exposure duration $T_{2}$ = 10${}^{4}$ sec.*” Note that a longer $T_{2}$ results in a lower MPE. In this case, it is implicit that exposures longer than 10${}^{4}$ seconds pose no additional hazard, so the MPE computed under this rule is, in effect, for indefinite viewing. The $T_{2}$ we will adopt is:

(A5) $$T_{2}=10^{4}\;\sec$$

(A6) $$C_{pupil\_area}=\pi \cdot {\left(\frac{0.7}{2}\right)}^{2}=0.385\;{\mathrm{cm}}^{2}$$

A plot of the Rule 2 MPE for a 0.9 × 0.9 deg field for wavelengths from 700 to 1,064-nm is shown in Figure A1.

#### Application of Rule 3

The following equations are used to compute the multiple-pulse MPE for thermal hazards:

(A7) $$\;\;\;\;\;\;\;\;\;\;\;\;\;\;MPE\left[\frac{W}{cm^{2}}\right]=\left(MPE_{single\;pulse}\cdot C_{P}\right)\cdot C_{pupil\_area}\;\;\;$$

(A8) $$\begin{array}{c} \;\;\;\;\;\;\;\;\;\;\;\;\;\;MPE_{single\;pulse}\left[\frac{W}{cm^{2}}\right]=\frac{1.8\cdot C_{A}\cdot C_{E}\cdot T_{1}^{-0.25}\times 10^{-3}}{C_{dilation\_factor}} \end{array}$$

(A9) $$C_{P}=\left\{\begin{array}{cc} n^{-0.25} & \mathrm{for}n\leq 40\\ 0.4 & \mathrm{for}n>40 \end{array}\right.$$

where n is the number of pulses. We will use 0.4 since we are computing the MPE for indefinite exposure times and therefore the number of pulses will always be over 40. $C_{P}$ will be reduced further by a factor of 0.5 since intentional viewing of the laser is being used (ANSI, 2014) (Section 8.3.5, p. 68).

(A10) $$\;\;\;\;\;C_{pupil\_area}=\pi \cdot {\left(\frac{0.7}{2}\right)}^{2}=0.385\;{\mathrm{cm}}^{2}$$

(A11) $$\;\;\;\;\;C_{A}=10^{0.002(\lambda -700)},\lambda =700-1,064\;\mathrm{nm}$$

(A12) $$\;\;\;\;\;C_{E}=\frac{\alpha_{x}+\alpha_{y}}{2\cdot \alpha_{min}}$$

where α are the x and y dimensions of the field size in mrad and $\alpha_{min}$ = 1.5 mrad.

(A13) $$\;\;\;\;\;\;\;\;\;\;\;\;\;\;T_{1}=5\times 10^{-6}$$

(A14) $$\begin{array}{ccc} & & \;\;\;\;\;\;\;\;\;\;\;\;\;\;\;MPE[W]\\ & & =C_{raster\;duty\;cycle}\cdot \left(MPE_{single\;pulse}\cdot C_{P}\right)\cdot C_{pupil\_area}\;\;\;\;\; \end{array}$$

### Explanation of multiplicative terms in the equations

$C_{A}$ compensates for reduced absorption of melanin for longer wavelengths. $C_{A}$ increases with wavelengths above 700-nm, which in effect increases the MPE and indicates reduced risk with increasing wavelength.

$C_{pupil\_area}$ : The pupil area factor is required because the system uses intrabeam viewing (i.e., all the light from the system enters the eye). Under normal illumination, when one measures the total power at the cornea in Watts, the pupil will allow only a fraction of the total power to enter the eye. Typical ANSI calculations take this into consideration. However, for intrabeam viewing, *all* the measured power enters the eye and so this fact needs to be factored in by multiplying the final MPE by a 7-mm pupil area in cm${}^{2}$. Adding this factor is more conservative as it reduces the MPE.

$C_{E}$ compensates for extended rectangular fields. The rationale is that larger fields distribute the light across a larger area, thereby reducing risk. The extended field size for Rule 2 is the raster size. The extended field size for Rule 3 is a small rectangle whose length is equal to the distance that the scanning spot traverses over time $t_{min}$ (see discussion of $t_{min}$ below) and whose height is equal to the minimum spot size, $\alpha_{min}$.

$C_{dilation\_factor}$ : This factor takes into consideration that when exposed to visible light, a human pupil will naturally constrict, offering some protection against hazard. MPEs are divided by this factor. Visible light has values for $C_{dilation\_factor}$ that are >1 and so the MPE is reduced. For wavelengths above 700-nm, this factor is equal to 1.

$C_{raster\;duty\;cycle}$ : This is the amount of time per frame that the laser is on in the AOSLO. This factor is practically important when taking measures of average power at the cornea. For a given average power, the raster duty cycle (equal to or less than 1) is inversely proportional to the peak pulse power. A duty cycle of less than 1 reduces the MPE. In the AOSLO system, the laser is typically only on during the forward sweep and the duty cycle is typically 0.4. However, for the experiments in this study, the 940- and 1,064-nm sources were on continuously.

$T_{1}$ is the thermal containment time $t_{min}$ and therefore sets the duration of the pulse and the length of the line of the pulsed segment for this type of scanning application (ANSI, 2014) (Table 6c, p. 85). There is additional guidance in the ANSI 2014 (Section 8.2.3, p. 66) regarding the meaning of $t_{min}$.
*Rule 3 applies only to MPEs for thermal injury, where all pulses delivered in less than $t_{min}$ are treated as a single pulse. (Rule 3 protects against subthreshold pulse-cumulative thermal injury.) For individual pulses or groups of pulses, either delivered within a time frame less than $t_{min}$, or when the inter-pulse spacing between pulses or pulse groups is less than $t_{min}$, these pulse structures are treated as a single pulse in applying this rule.... NOTE—For high pulse repetition rates where multiple pulses occur in a time frame less than $t_{min}$, pulse energies delivered within those time frames are summed directly. It is assumed that the energy within $t_{min}$ acts as if it were delivered in a single pulse.*

Based on the text above, it is clear that ANSI provides no guidance on hazards or potential hazards from short-pulsed lasers ($t<t_{min}$) in the near infrared. In our case, the thermal hazard is the same whether the scanning beam comprises short pulses or is continuous. The fact that the light in the AOSLO is raster-scanned, however, makes the beam behave as a pulsed source, and this is what is considered in the Rule 3 calculation. Each “pulse” comprises a short line, the length of which is determined by the thermal containment time $t_{min}$ and the frequency of which is set by the frame rate of the scanning system.

It might appear from the calculation that the multiple-pulse MPE ignores the fact that there are many pulses occurring across the area of the extended field. In fact, the single-pulse MPE is multiplied by the duty cycle of the pulse to reflect the fact that the pulse comprises only a brief period of time per frame, but then the MPE, which is the total power measured at the eye, is divided by the same duty cycle to reflect the fact that there are multiple pulses occurring across the extended field. These two factors cancel each other.

A plot of the Rule 3 MPE for a 0.9 × 0.9 deg field for wavelengths from 700 to 1,064-nm is plotted on Figure A1.
